# Influence of Filler Type and Rheological Properties of Asphalt Mastic on the Asphalt Mastic–Aggregate Interaction

**DOI:** 10.3390/ma16020574

**Published:** 2023-01-06

**Authors:** Guangxun E, Jizhe Zhang, Quanjun Shen, Ping Ji, Jing Wang, Yushuai Xiao

**Affiliations:** 1 Shandong Key Laboratory of Highway Technology and Safety Assessment, Shandong Hi-Speed Group Co., Ltd., Jinan 250101, China; 2 School of Qilu Transportation, Shandong University, Jinan 250100, China; 3 Shandong Hi-Speed Engineering Consulting Group Co., Ltd., Jinan 250100, China

**Keywords:** filler, asphalt mastic, interfacial bond strength, asphalt–aggregate interaction, moisture damage

## Abstract

The asphalt mastic–aggregate interaction plays an important role in the overall properties of asphalt mixtures and their durability in service in flexible pavements. This paper aims to study the influence of the physico-chemical features of fillers and the rheological properties of asphalt mastics on the bonding behavior between asphalt and aggregate, and the interfacial deterioration mechanism when subjected to static water immersion and pressured water immersion. It was found that the filler type (limestone powder, basalt powder, and granite powder) had a certain influence on the complex modulus of asphalt mastics, and its pore volume and specific surface area had significant effects on the phase angles and permeability of asphalt mastics. The effect of water pressure can accelerate the deterioration of bond strength of the asphalt mastic–aggregate interface in the short term, indicating that the dynamic water pressure generated by the driving load promotes the water damage process in asphalt pavements. In comparison, the residual bond strength ratio of the granite–asphalt mastic aggregate was the highest, while its bond strength was lower than that of the interface between limestone–asphalt mastics and limestone aggregate. This demonstrated that a low asphalt mastic complex modulus and a high phase angle are helpful in improving the durability of asphalt mixtures subjected to static and pressured water immersion conditions.

## 1. Introduction

Owing to the advantages of smooth surfaces, driving comfort, low noise, and easy maintenance, flexible asphalt pavements have become the main pavement type for high-grade highways in most countries [1,2]. The asphalt mixture used in flexible asphalt pavements is a multiphase composite, which is composed of asphalt binder, filler, voids, aggregates of different sizes, and the asphalt–aggregate interface [3]. Therefore, it is normally considered to be a heterogeneous material and its properties can be strongly influenced by different material variables and their proportions.

In previous research, pavement engineers and researchers recognized that the asphalt mastic and the asphalt mastic–aggregate interface strongly determine the overall performance of the asphalt mixtures and their durability in service life [4]. The asphalt mastic in the asphalt mixture is the cementing component and consists of bitumen and fine aggregate, and its composition and properties directly affect the road performance of asphalt mixtures. The physico-chemical properties of the mineral fillers incorporated have a significant impact on the performance of asphalt mastics. Zhang et al. used oxide analytical reagents to represent mineral aggregate fillers and to study their effects on the properties of asphalt mastics and the interfaces. It was found that the effects of calcium oxide (CaO) were greater than those of silicon dioxide (SiO_2_) due to the stronger interaction between asphalt binder and CaO [5]. Based on the research on the mesoscopic properties of mineral fillers, Lv et al. found that physical properties such as mineral filler fineness and mesoscopic gradation had a certain correlation with the mesoscopic strength of asphalt mastics [6]. Barra et al. analyzed the influence of the properties of limestone mineral powder and granite mineral powder on the softening point, penetration, and adhesion of asphalt mastic [7]. White studied the shear creep response of an airport asphalt mastic and the results indicated that two types of asphalt mastics had different properties due to different dusts [8,9]. Compared with the size of the filler, its shape, surface texture, specific surface area, and mineral components had more significant effects on the properties. Based on the microstructure of fillers, Geber et al. analyzed the effects of the particle size distribution, microscopic morphology, mercury intrusion porosity, specific surface area, and hydrophobicity of limestone powder and dolomite powder on the rheological properties of asphalt mastics [10]. The particle size, hydrophobicity, and content of fillers have significant effects on the rheological properties of asphalt mastics.

In recent years, more studies have also been carried out to investigate the adhesion of the asphalt mastic–aggregate interface. In general, four mechanisms have been adopted to explain the adhesion between asphalt mastic and mineral aggregate: surface energy, chemical reaction, molecular orientation, and mechanical contact [11]. Yi et al. studied the influence of different factors on the adhesion of the asphalt–aggregate interaction based on the surface energy theory and showed that the measured surface energy using an AFM method can represent the surface characteristics of materials [12]. Based on the molecular orientation theory, Huang et al. found that the fundamental cause of adsorption of asphalt on the aggregate surface was the polarity of the asphalt and aggregate and that the compositions of asphaltene and gelatine greatly determined adhesion [13]. Using digital imaging techniques and an abrasion method, Kuang et al. studied the macroscopic changes on the particle surface of limestone and granite before and after treatment and their influences on interfacial adhesion between asphalt and limestone/granite. It was found that the interfacial bonding between asphalt/mastic and aggregate in asphalt mixtures is mainly attributed to the micro/meso-physical and chemical actions in the vicinity of the interface [14].

Most importantly, factors such as water, temperature, environmental conditions, and other incorporated recycled waste, such as waste oil and industrial waste powder, also have important effects on the rheology and durability of asphalt mixtures [15,16,17]. Because, in practice, asphalt pavements have a long service life, they suffer from different degrees of defects. Water/moisture damage is a common early defect that can lead to the loosening and peeling of aggregates, which seriously affects the pavements service performance and shortens its service life [17,18,19]. Under the dual action of water and driving traffic load, the asphalt mastic–aggregate interface may become the weakest zone of the asphalt mixture, even after initially exhibiting a good interfacial bond strength.

The stripping potential of asphalt mixtures is usually evaluated based on the cohesion bond between the binder and aggregates. It is often caused by the loss of the mastic–aggregate bond and results in poor durability of asphalt mixtures. This study, therefore, aimed to experimentally assess the correlation between the physico-chemical features of fillers, the rheological properties of asphalt mastics, and the asphalt mastic–aggregate interaction before and after static and dynamic water attack. Three types of fillers and aggregates that are normally used in asphalt mixtures (limestone, basalt, and granite) were chosen and characterized. The influence of fillers on the rheological properties of asphalt mastics was characterized using a dynamic shear rheometer (DSR). The direct bond strength of the asphalt–aggregate interface as a key property indicator was measured and evaluated. The water attack testing was conducted by increasing the water pressure. Understanding the bond strength development and the deterioration of the asphalt mastic–aggregate interface will help to provide scientific guidelines for the design of asphalt mixtures.

## 2. Materials and Experimental

### 2.1. Materials

#### 2.1.1. Bitumen

The bitumen used as a binder in the asphalt mixtures was a type of AH-70^#^ road petroleum (produced by a local company in Shandong, China). It was an unmodified type with a penetration value of 60–70 (according to ASTM D5, it was 60/70 grade). Its properties, which were in accordance with Chinese test methods, are listed in Table 1.

#### 2.1.2. Filler

The mineral powders used as fillers in the asphalt mixtures in this study were limestone mineral powder (LP), basalt mineral powder (BP), and granite mineral powder (GP), which were sourced from these three types of minerals. These mineral particles, which were less than 4.75 mm in size, were ground in a laboratory mill for 2 min and then passed through a 0.075 mm sieve to obtain powder. The densities of the three kinds of mineral powder were measured using a pycnometer (Chinese standard, T0352) and the results are listed in Table 2.

The particle size distributions of these three fillers, measured using a laser diffraction technique (Tester of LS230), are shown in Table 3. *D*_10_, *D*_50_, and *D*_90_ represent the minimum particle size with a pass rate of 10%, 50%, and 90%, respectively. It was found that the *D*_10_, *D*_50_, and *D*_90_ of LP exhibited a relatively smaller size than those of the other BP and GP fillers. This indicates that LP was finer than the others, and BP and GP had similar sizes.

The mineral composition of the mineral powder has an important influence on its chemical reaction with bitumen [20]. Table 4 shows the oxide chemical components of the three mineral fillers measured by X-ray fluorescence (XRF) (Rigaku, Tokyo, Japan, Supermini200). It can be seen that the chemical composition of the three mineral powders was significantly different. The main component of LP was CaO and its content was about 83% by mass, while the content of SiO_2_ was the lowest (5.8%) compared to the others. The LP is, therefore, considered as an alkaline mineral. As regards the basalt powder, its main chemical components were SiO_2_, Al_2_O_3_, Fe_2_O_3_, and CaO, and the SiO_2_ content was 46% (mass fraction), which makes it a neutral mineral powder. The main components of the granite powder were SiO_2_ and Al_2_O_3_, and its SiO_2_ content was 64% (by mass). Thus, it is regarded as an acidic mineral powder. These chemical components cause the powders to have noticeably different chemical reactions with bitumen.

**Figure 1:**
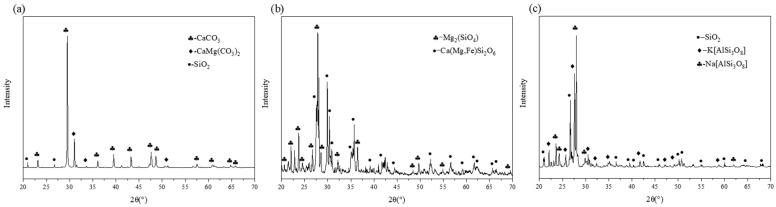
XRD diffractions of mineral fillers: (**a**) limestone powder; (**b**) basalt powder; (**c**) granite powder.

In order to detect the mineral crystals in the three kinds of fillers and then to identify their influence on the performance of asphalt mastics and interfaces, their diffraction patterns were obtained by XRD (Rigaku SmartLab), and the mineral crystal analysis was carried out using the HighScore Plus software. The results are shown in Figure 1. It was found that (1) the main mineral crystalline components of LP were calcite (CaCO_3_), dolomite (CaMg(CO_3_)_2_), and a small amount of quartz (SiO_2_). Its acidic polar group forms a stable double bond structure, and the chemical bond is not easily destroyed when exposed to water. (2) The main mineral components of BP were olivine (Mg_2_(SiO_4_)) and pyroxene (Ca(Mg,Fe)Si_3_O_6_). (3) The main mineral components of GP were quartz (SiO_2_), albite (Na[AlSi_3_O_8_]), and potassium feldspar (K[AlSi_3_O_8_]). The acidic quartz is normally weakly bonded with bitumen and does not easily form a strong bonding interface. 

#### 2.1.3. Aggregates

The cylindrical aggregate samples, made of limestone, basalt, and granite, were prepared to a size of Φ20 × 20 mm. First, a drill with an inner diameter of 20 mm was used to take a core of a large volume of the aggregate mineral, and then a high-precision double-sided cutting machine was used to cut the cylindrical core sample, ensuring a height of 20 mm. Thereafter, both surfaces of the sample were polished with sandpaper to ensure a similar texture. Finally, the aggregate samples were washed in boiling water at 100 °C and dried. Their physical properties are listed in Table 5. The technical indicators of the three aggregates met the requirements of the Chinese Specification JTG F40-2004 “Technical Specification for Highway Asphalt Pavement Construction”.

### 2.2. Preparation of the Specimens

#### 2.2.1. Design of Asphalt Mastics

In the study, asphalt mastic was defined and designed as a mixture of bitumen and calcareous or siliceous fillers. In order to minimize the effect of the mineral powder volume on the asphalt mastics, they were designed with a ratio of bitumen to filler = 1:1 by volume [21,22,23]. The filler was first dried at 150 °C for 3 h. Then, the bitumen was heated in a furnace at 150 °C for 1 h. After placing the filler in a vessel with a temperature of 150 °C, an enhanced stirring process was conducted to mix the bitumen and filler homogenously at a speed of 1000 rpm for 30 min. The three types of asphalt mastics (LM, BM, and GM) were then obtained in order to carry out the following tests.

#### 2.2.2. Design of the Asphalt Mastic–Aggregate Interface Specimens

**Figure 2:**
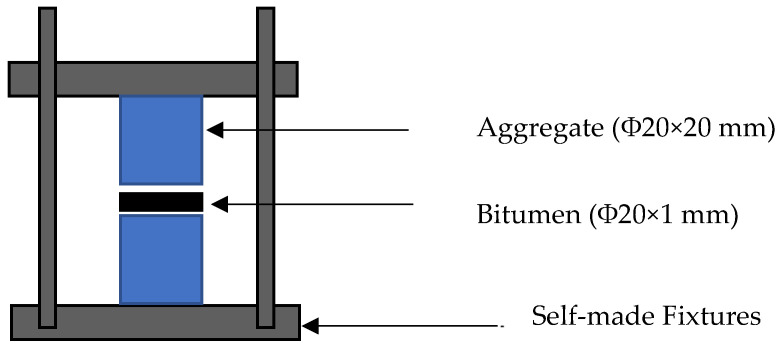
Schematic of the preparation of asphalt mastic–aggregate interface specimens.

In order to quantitatively measure the adhesion of the asphalt mastic–aggregate interface, the asphalt mastic–aggregate interface specimens were prepared as shown in Figure 2. The mechanical failure tests were conducted by controlling the asphalt mastic thickness within 1 mm. The sample preparation processes included the following: (1) Installing a mold with the aggregates; (2) aligning the cylindrical aggregates (Φ20 × 20 mm) in self-made fixtures; (3) loading the asphalt mastic in between two pieces of aggregates; (4) controlling the thickness of the asphalt mastic within 1 mm; (5) demolding the asphalt–aggregate interface specimen; (6) removing the extra asphalt mastic around the interface. Then, the bond strength of the interface was tested using a testing machine. In this study, a 1 mm spacer was used to control the thickness of the asphalt mastic, and three types of mastics and three aggregates were used to prepare nine groups of asphalt mastic–aggregate interface specimens. The specific specimen designs and names are shown in Table 6. 

### 2.3. Experimental Tests

#### 2.3.1. Rheological Testing of Asphalt Mastics

- A dynamic shear rheometer (DSR) was employed in this study to evaluate the rheological properties of the asphalt mastics, focusing on the influence of fillers. In this test, mastic specimens with a 2 mm thickness were prepared and sandwiched between two flat plates with a diameter of 8 mm. One of the two plates was fixed and the other one was oscillated back and forth around the central axis at a certain angular velocity. The mastic specimens were tested at a temperature interval of 10 °C in the range of 30–60 °C and the scanning rate was 10 rad/s. According to the curves, the complex shear modulus G*, phase angle δ, and rutting factor (G*/sin δ) of the asphalt mastics at different temperatures were obtained. The results were employed to evaluate high-temperature performance of the asphalt mastics at moderate temperatures;
- The bending-beam rheometer (BBR) test was employed to characterize the low-temperature cracking resistance of the asphalt mastics. In this research, the BBR test was performed at −6 °C, −12 °C, and −18 °C. During testing, a constant load of 980 ± 50 mN was added in the middle of the mastic beam for 240 s. The deflection was automatically recorded in order to calculate the creep stiffness (S) and m-value (m). In order to resist thermal cracking at low temperatures, the creep stiffness (S) and the m-value must meet certain requirements.

#### 2.3.2. Bond Strength Testing of the Asphalt Mastic–Aggregate Interface

**Figure 3:**
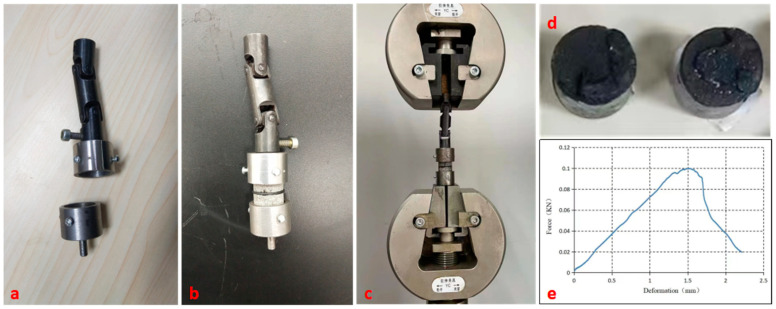
Testing of asphalt mastic–aggregate interface specimens: (**a**) holding fixture; (**b**) specimen fixed by holding fixture; (**c**) specimen fixed into universal testing machine; (**d**) specimen failure surface; (**e**) loading curve.

As shown in Figure 3a–e, a universal testing machine with an accuracy of 1 N was employed to measure the maximum force using a deformation-controlled model. Its loading speed was 0.01 mm/s. The bond strength of the interface was then calculated by:

(1) $$f_{t}=\frac{F}{S}$$

where *F* is the maximum failure force; *S* is the interfacial area; *f_t_* is the bond strength of the interface.

#### 2.3.3. Water Absorption of Asphalt Mastics

In order to understand the diffusion behavior of water in the asphalt mastics under normal temperature and pressure, the moisture absorption rates of the asphalt mastics subjected to different water immersion periods were measured using a gravimetric method, and the change principle of the water contents in the asphalt mastics was analyzed by measuring the change in its mass against time [24]. The specific testing processes were given as follows: (1) A customized aluminum plate mold (the mass is *m*_0_) was used to prepare an asphalt mastic film of 50 mm × 50 mm × 0.3 mm; (2) an analytical balance was used to weigh the aluminum plate and the asphalt mastic before water immersion (the mass is *m*_1_); (3) the samples were immersed in distilled water and removed at regular intervals; (4) the water was wiped off with filter paper and the mass m_t_ of the asphalt mastic plus the aluminum plate was measured. The test results were recorded after soaking times of 1 h, 4 h, 12 h, and 24 h…

The moisture absorption rate (*M_t_*) of the asphalt mastic at the time of immersion *t* was then calculated by:

(2) $$M_{t}=\frac{m_{t}-m_{1}}{m_{1}-m_{0}}\times 100\%$$

#### 2.3.4. Water Attack Testing of the Asphalt Mastic–Aggregate Interface

In order to investigate the influence of different water conditions on the bond strength of the asphalt mastic–aggregate interface, the temperature (10~40 °C), static water immersion time (7 d, 14 d), and water pressure action time (12 h, 24 h) were assessed to test their influence on the mechanical properties of the interface. As calculated by the effect of standard axle load and average velocity, the variation range of the pore water pressure of the asphalt pavement surface layer is generally 0.20~0.57 MPa [25,26]. In this study, a self-designed pressure device was used to simulate the water pressure. Its pressure was 0.5 MPa.

## 3. Results and Discussion

### 3.1. Physical Features of Three Types of Filler

**Figure 4:**
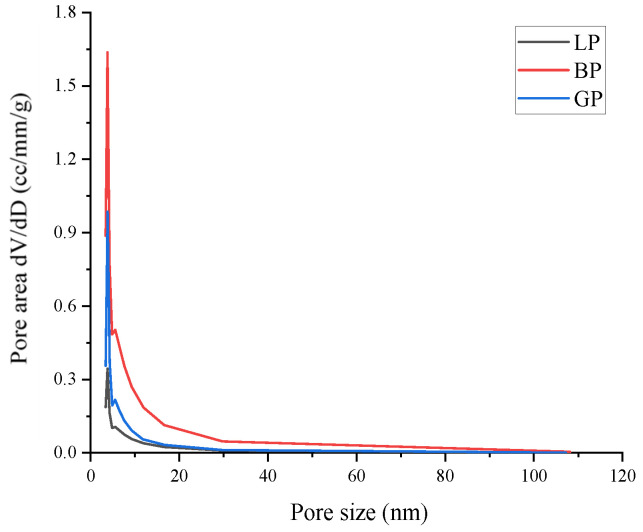
Pore size distribution of mineral powders by BET.

The Brunauer–Emmett–Teller (BET) method was applied to calculate the specific surface area of the fillers according to nitrogen adsorption isotherm measurements. Using the Barrett–Joyner–Halenda (BJH) model, the pore size distributions of the fillers were derived from the adsorption branches of the isotherms. The pore volume distributions of the mineral fillers as assessed by physical adsorption under high vacuum conditions (measured using Micromeritics ASAP2020 PLUS) are shown in Figure 4. The pore size distribution ranges of LP and GP were roughly similar, ranging from 4 to 20 nm, while the pore size distribution of BP was relatively wider, ranging from 4 to 40 nm. The BJH pore volume, the average pore size, and BET specific surface area of the fillers are listed in Table 7. It can be observed that the pore volumes of LP and GP were 0.007 cm^3^/g and 0.009 cm^3^/g, respectively, which were obviously lower than that of BP with 0.034 cm^3^/g. The average pore sizes of BP and GP were similar, i.e., 3.810 nm and 3.819 nm, respectively, and the average pore size of LP was slightly smaller at 3.059 nm. According to the specific surface area calculated by BET, it can be seen that the specific surface area of LP was the smallest (1.899 m^2^/g), while the specific surface area of BP was the largest (9.008 m^2^/g). Moreover, the specific surface area of GP (3.42 m^2^/g) was between LP and BP. As previously reported, the differences in the pore volume and specific surface area of the mineral powders certainly affect the selective absorption of the asphalt component according to the filler, and then affect the performance of the asphalt mastic and mixture [27].

**Figure 5:**
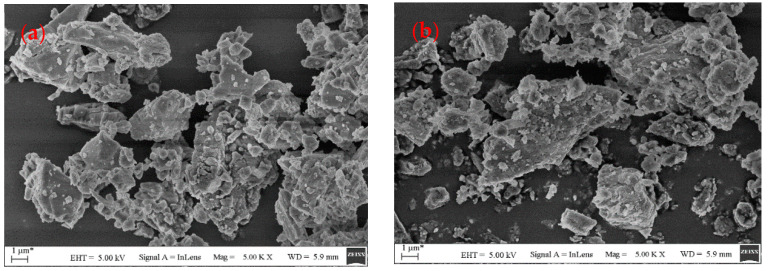

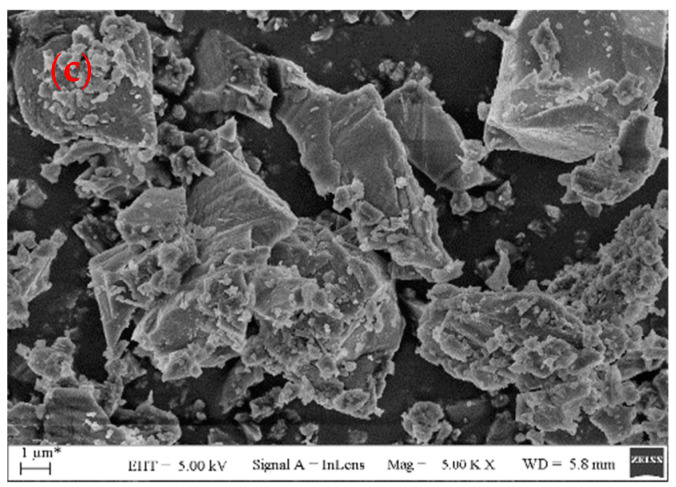
Morphology of mineral filler surface: (**a**) LP; (**b**) BP; and (**c**) GP.

The microscopic morphology of the mineral fillers directly affects the selective absorption of asphalt, resulting in changes in the rheological properties of asphalt mastics. The micromorphology of the fillers as assessed using SEM (Tescan, Brno, Czech Republic, Vega 3) is shown in Figure 5. It can be observed that the particle morphology and surface texture of the three mineral fillers were different. The LP particles were relatively smooth, without obvious edges and corners, and the particle size was relatively fine, with a small amount of fine flocculent particles attached to the surface of the coarse ones. BP had a relatively complex surface texture, a rough texture, a large number of holes, and small channels. The GP particles had a clear outline, more polygonal particles, obvious edges and corners, and no obvious holes in the particles. It can be hypothesized that BP absorbs light components of bitumen into the particles and this influences the rheological response. 

### 3.2. Rheological Properties of Asphalt Mastics at High Temperature

**Figure 6:**
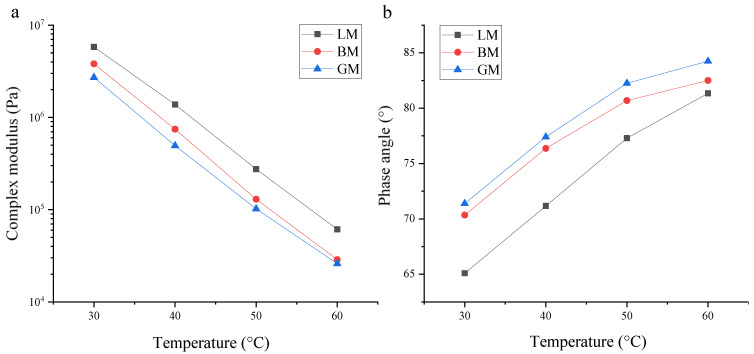
Rheological properties of asphalt mastics: (**a**) complex modulus and (**b**) phase angle.

A dynamic shear rheology tester (DSR) was employed to conduct a temperature sweep test in order to evaluate the rheological properties of the asphalt mastics. Their complex modulus and phase angle curves are shown in Figure 6. The complex modulus is a measure of the total resistance of a material when it is repeatedly sheared and deformed, and the higher its value, the stronger the ability of the asphalt mastic to resist deformation [28]. At the same temperature, the complex modulus of LM was the highest, indicating that its high-temperature deformation resistance is strong, which may be related to the particle size of LP and its chemical reaction with bitumen. The complex modulus of BM was second and that of GM was the lowest, which may be due to the difference in the pore volume and specific surface area. With the increase in temperature, the asphalt mastic changed from a viscoelastic state to a viscous fluid state, which increased the viscous components in the asphalt mastics and increased the phase angle. Under the same temperature, the phase angle of LM was the lowest, the phase angle of GM was the highest, and the phase angle of BM was in between. The LP had the smallest particle size and better dispersibility in the bitumen, resulting in LM exhibiting the lowest phase angle. The particle sizes of BP and GP were similar, but their specific surface areas and pore volumes were significantly different, and the pore volume of BP was three times that of GP. BM had the most structural bitumen and the least free bitumen, resulting in a highly elastic composition. The phase angle of BM was lower than that of GM. From these results, it can be seen that both the chemical composition and morphology of the fillers determine the rheological properties. First, the alkaline filler, LP, exhibited a very good chemical behavior with bitumen and this resulted in its high complex modulus, low phase angle, and even, smooth morphology. The porous features of BP enhance its complex modulus.

**Figure 7:**
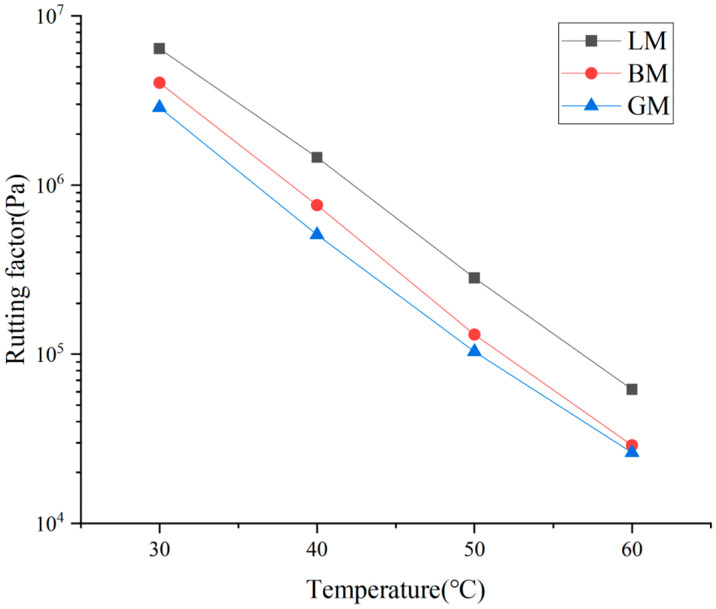
Rutting factors of asphalt mastics.

The rutting factor G*/sinδ, which is commonly used in the Superpave specification, characterizes the long-term deformation ability of asphalt mastic, with a high value indicating a high ability to permanently deform. Figure 7 shows the rutting factors of the different types of asphalt mastics. At the same temperature, the order of the rutting factor was LM, BM, and GM, which was related to the particle size, pore volume, and surface area. The particle size of LP was the smallest, which made it more dispersed in bitumen, so LM had better resistance to permanent deformation. The specific surface area and pore volume of BP were three times those of the granite mineral powder, so that the permanent deformation resistance of BM was higher than that of GM.

### 3.3. Rheological Properties of Asphalt Mastics at Low Temperature

**Figure 8:**
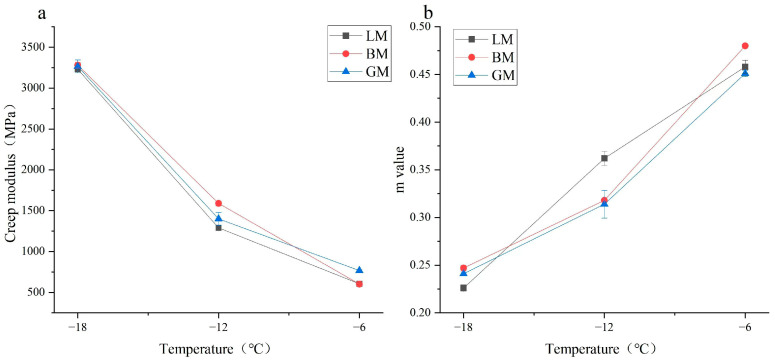
Rheological properties of asphalt mastics at low temperatures: (**a**) stiffness modulus and (**b**) creep rate.

The creep stiffness modulus S as assessed using the BBR characterizes the low-temperature performance of asphalt mastics. The higher the S value, the worse the low-temperature cracking resistance of the mastic. The creep rate m characterizes the change rate of the stiffness of the mastic with time, with high values indicating low deformation [29]. The results of the stiffness modulus S and m values of asphalt mastics are shown in Figure 8. It can be seen that the S of the three asphalt mastics decreased with the increase in the temperature, and the m increased with the increase in the temperature. This shows that, with the decrease in the temperature, the low-temperature cracking resistance of the three asphalt mastics reduced. The S and m values of the three mastics were not obviously different, indicating that the type of mineral filler had relatively less effect on the low-temperature performance of asphalt mastics. BP had the largest specific surface area, and it was shown to adsorb more light bitumen components. When the content of structural components increased, it resulted in the highest S value for BM at −18 °C and −12 °C. The slope m-value was introduced. A low slope value indicates a lower capacity to endure the stresses produced at low temperatures. In Figure 8b, BM at different temperatures (−6, −12, and −18) did not follow the same trend as LM and BM. This might be because of porous physical features of BP resulting in a different low-temperature resistance. The S value of LM was relatively low, the m value was high, and the cracking resistance was good, which was also related to its small specific surface area and pore volume.

### 3.4. Water Diffusion of Asphalt Mastics

**Figure 9:**
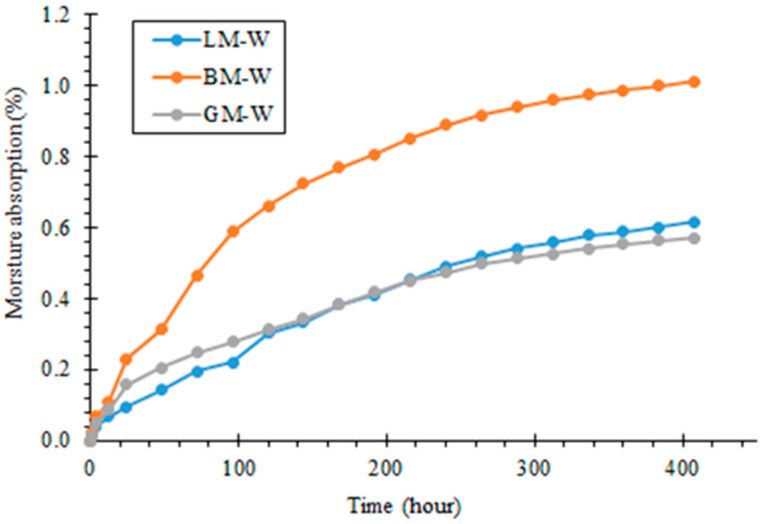
Moisture absorption curves of asphalt mastics.

In order to study the diffusion characteristics of water in asphalt mastics subjected to normal temperature and pressure, the moisture absorption curves of asphalt mastics under different immersion times were obtained using a gravimetric method. The results are shown in Figure 9. With the prolongation of the immersion time, water continuously diffused into the asphalt mastics and gradually became saturated. After soaking for 384 h, the change in the moisture absorption rate of the asphalt mastic tended to be gentle and essentially reached saturation. When exposed to the condition of water immersion, the moisture absorption rate of BM was the highest, followed by LM and GM. This could be related to the pore features of BP, i.e., a high pore volume and surface area.

The moisture absorption rate represents the moisture absorption characteristics of different asphalt mastics. To understand the diffusion properties of water in the asphalt mastic, the moisture absorption curves were fitted based on the Fick diffusion model using Equation (3) [30], and the diffusion coefficients of D are listed in Table 8. It can be seen from the fitting results under the same conditions that the order of diffusion coefficients of water in the three types of mastics was BM, LM, and GM. Because BP had the largest specific surface area and pore volume, and had a fluffy structure, many pores, and small channels in the particles, BM had the ability to absorb more water. LM and GM had similar diffusion coefficients.

(3) $$\frac{M_{t}}{M_{\infty }}=1-\sum_{n=0}^{\infty }\frac{8}{{\left(2n+1\right)}^{2}\pi^{2}}e^{\frac{-D{\left(2n+1\right)}^{2}\pi^{2}t}{l^{2}}}$$

where *n* is a natural number, *D* is the diffusion coefficient, *l* is the thickness of the sample, and *M∞* is the equilibrium moisture absorption rate.

### 3.5. Bond Strength of the Asphalt Mastic–Aggregate Interface

#### 3.5.1. Influence of Temperature

**Figure 10:**
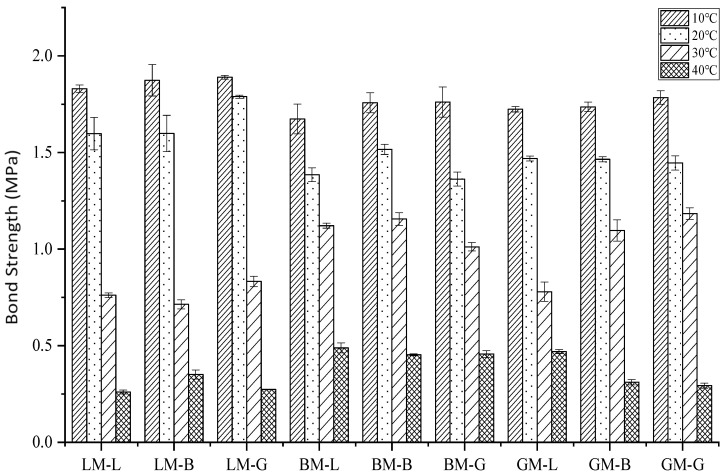
Bond strengths of asphalt mastic–aggregate interfaces at different temperatures.

The bond strength results of the asphalt mastic–aggregate interfaces at different temperatures are shown in Figure 10. At 10 °C and 20 °C, the LM–L, LM–B, and LM–G interfaces had the highest bond strengths, compared to the other interfaces. For 30 °C and 40 °C, the BM–L, BM–B, and BM–G interfaces had the highest bond strengths. This shows that the interface bond strengths of the asphalt mastics were strongly determined by temperature and the mastic type. When increasing the temperature, the interface strength decreased gradually. The effect of temperature on the interface bond strength of the interfaces exhibited similar trends. Under 20 °C, the bond strength of the three interface combinations using LM was greater than that of BM and GM.

**Figure 11:**
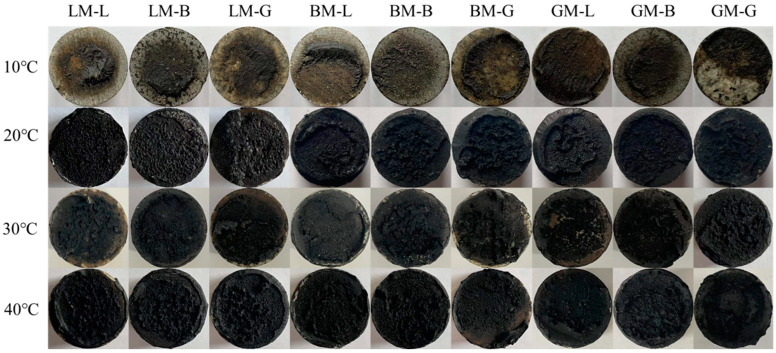
Failure modes of asphalt mortar–aggregate interfaces at different temperatures.

Fracture interfaces are illustrated in Figure 11. The failure modes of the asphalt mortar–aggregate interface were divided into two types: cohesion failure inside the asphalt mastic and adhesion failure at the asphalt mastic–aggregate interface. Under 20 °C, the failure mode was mainly the cohesion failure of the asphalt mastic–aggregate interface, indicating that the LM–aggregate interface had the best adhesion in the dry state and at a relatively low temperature. With the increase in temperature, the failure mode gradually changed from cohesion failure to adhesion failure. The bond strength of the specimen was mainly dominated by the adhesive strength of the asphalt mastic. The bond strength of the specimen prepared using BM was the highest, indicating that the adhesive strength of the BM system was higher, which was related to the larger pore volume, specific surface area, and surface morphology of BP. Moreover, the structural component of bitumen in the asphalt mastic can produce good adhesion with coarse aggregates. 

#### 3.5.2. Influence of Water Immersion without Pressure

**Figure 12:**
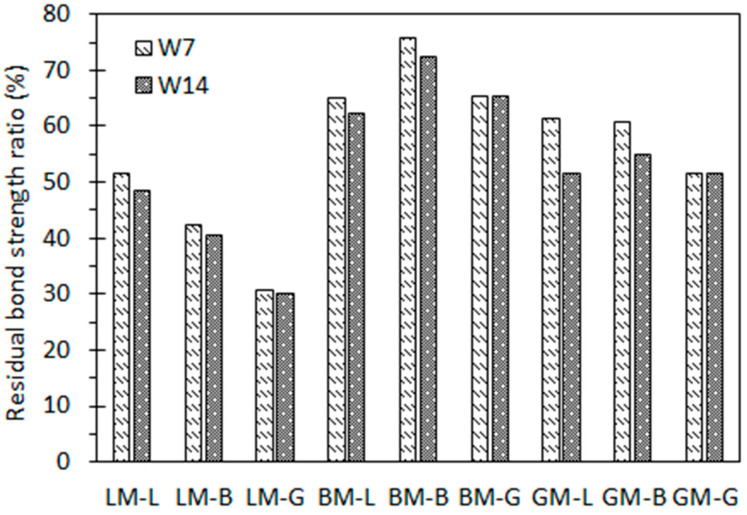
Residual bond strength ratios of asphalt mortar–aggregate interfaces under 7-day and 14-day water immersion.

**Figure 13:**
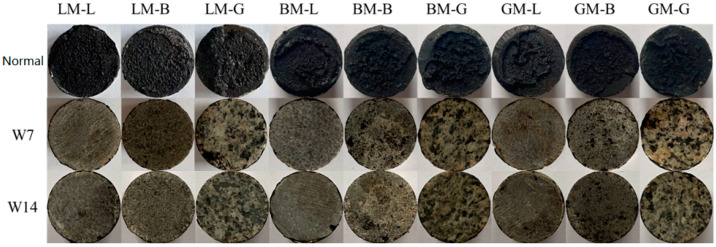
Failure of asphalt mastic–aggregate interface under water immersion.

Figure 12 shows the residual bond strength ratios of asphalt mastic–aggregate interfaces under immersion in water for 7 days (W7) and 14 days (W14) without pressure. The residual bond strength was defined as the ratio between the bond strength before water immersion and after water exposure. It can be seen that when subjected to water immersion, all interfacial bond strengths decreased. This also gradually decreased with the extension of the immersion period from 7 days to 14 days. For the same immersion period, the residual bond strength ratios of BM–L, BM–B, and BM–G were between 62% and 76%, which were higher than those of the other mastic–aggregate interfaces. This may be attributed to the higher content of structural asphalt in BM, and the larger pore volume of basalt helps to produce stable mechanical interlocking with asphalt, while the difference in the diffusion coefficient between the aggregate and asphalt does not play a dominant role. For the same asphalt mastic, the mastic–granite aggregate interfaces had relatively lower residual bond strength ratios compared to other two types of aggregates. This is because the interface formed by the weakly acidic mineral components of the granite aggregate and the asphalt mastic peels off easily when exposed to water, resulting in a rapid attenuation of its strength. Moreover, it was noticed that after water immersion, the residual bond strength ratios of the LM–aggregate interfaces were lowest, indicating that the water attack was more serous in the LM–aggregate interfaces. The specimens prepared from LM did not obtain the ideal residual bond strength ratios, which may be affected by two factors: (1) The specimens prepared from LM (LM–L, LM–B, and LM–G) were in a dry state. Their bond strengths were the highest, resulting in the highest initial bond strength values; (2) the small pore volume and specific surface area of LP result in the mechanical interlocking with asphalt being weak, and thus, it can be eroded by water. Figure 13 shows the failure images of each asphalt mastic–aggregate interface when subjected to water immersion. It can be seen that the interface was completely damaged via adhesion failure, indicating that after immersion in water for 7 d and 14 d, the water can reach the asphalt mastic–aggregate interface and the interface is adhesively damaged.

#### 3.5.3. Influence of Water Pressure

**Figure 14:**
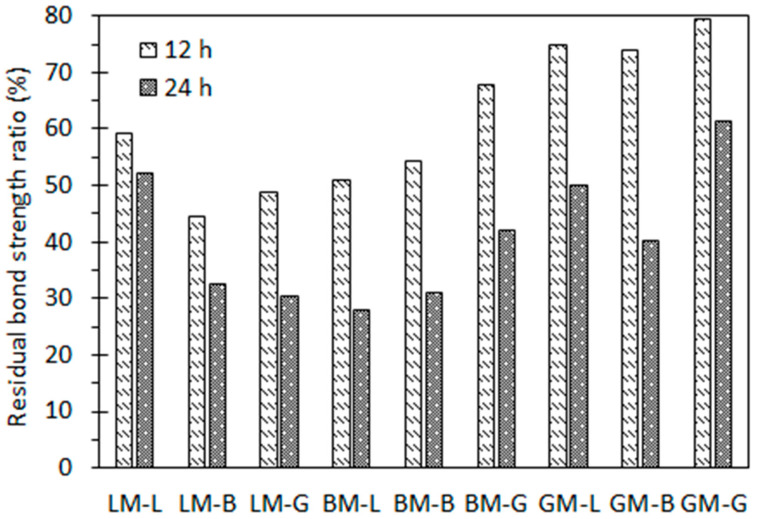
Residual bond strengths of asphalt mastic–aggregate interfaces under water pressure.

**Figure 15:**
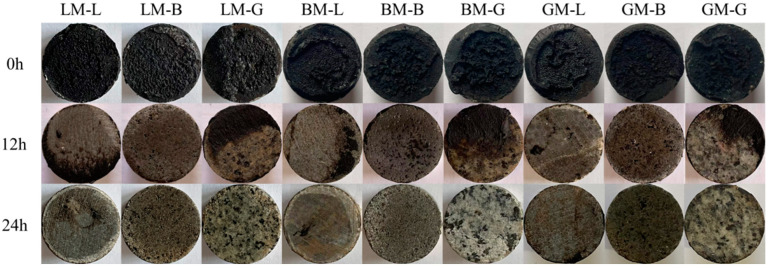
Failure of asphalt mortar–aggregate interfaces under water pressure.

Figure 14 shows the residual bond strength ratios of the asphalt mastic–aggregate interfaces subjected to a water pressure of 0.5 MPa for 12 h and 24 h. It can be seen that with the prolongation of the water pressure action time, the residual bond strength ratio of each type of interface gradually decreased. For comparison, the residual bond strength of the GM–aggregate interfaces (GM–L, GM–B, and GM–G) was the highest, which may be due to the dominant effect of the low diffusion coefficient of GM under the action of water pressure. Figure 15 shows the interface failure images of each interface after water pressure for different times. It can be seen that, in the absence of water pressure (0 h), interface cohesion failure occurred. When water reached to the bonding interface with pressure (12 h and 24 h), interface failure developed from cohesion failure to adhesion failure.

It can be seen from Figure 12 and Figure 14 that the residual bond strength ratios of the asphalt mastic–interfaces under water pressure for 24 h were even lower than those under static water immersion for 14 days. The water pressure can accelerate the attenuation of the asphalt mastic–aggregate interface bond strength, indicating that the hydrodynamic pressure generated by the traffic load can promote the water damage process of the asphalt pavement. This is also consistent with the conventional understanding that alkaline limestone has strong adhesion and acid granite has weak adhesion. Pressure immersion can accelerate the deterioration process of asphalt mastic–aggregate specimens as a result of water. However, by comparison, it was found that the attenuation law of the interfacial bond strength of the asphalt mastic–aggregate interface under water pressure was different under static water immersion. It can be seen that the water stability of the interface specimens was related to the properties of the aggregates and fillers and the diffusion behavior of the asphalt mastics. 

## 4. Conclusions

The influence of the physico-chemical features of fillers and the rheological properties of asphalt mastics on the bonding behavior between asphalt and aggregate, and the interfacial deterioration mechanism subjected to static water immersion and pressured water immersion was tested experimentally and evaluated. The main findings are as follows:

- The mineral filler type influenced the complex modulus and low-temperature performance index of the asphalt mastic, and the difference in pore volume and specific surface area changed the content of the structural asphalt components in the asphalt mastics, thereby affecting the phase angle. The specific surface area of the basalt filler was the largest, resulting in a high content of structural asphalt components, and its mastic had a relatively higher stiffness modulus at −18 °C and −12 °C. The chemical composition of the filler was the primary factor in determining rheological behavior and the morphology was the secondary factor in influencing the properties of asphalt mastics;
- When exposed to the water immersion, the moisture absorption rate of the basalt mastic was the highest, followed by the limestone and granite mastics, and the granite mastic had the lowest diffusion coefficient. This may be related to the pore features of the basalt filler, which had a high pore volume and surface area;
- The alkaline limestone aggregate exhibited strong initial adhesion with bitumen and the acid granite aggregate exhibited weak adhesion. When the complex modulus of asphalt mastic was high and its phase angle was low, it resulted in a good initial bonding behavior with aggregate in the dry state. However, this does not indicate that such an interface between alkaline limestone aggregate and asphalt mastic exhibits good durability, e.g., against water attack;
- Static and pressured water immersion conditions can accelerate the deterioration process of asphalt mastic–aggregate interfaces. For the three asphalt mastics, when the complex modulus of the asphalt mastic was low and its phase angle was high, the durability of asphalt mixtures subjected to static and pressured water immersion conditions improved. The asphalt mastic with the acid granite filler initially exhibited relatively weak adhesion in the asphalt mastic, but it showed good water attack resistance between the asphalt mastic and coarse aggregate;
- The deterioration mechanism of the interfacial bond strength of the asphalt mastic–aggregate interface under static water immersion was different from pressured water immersion. It was found that the water stability of the asphalt mastic–aggregate interface was strongly related to the properties of the aggregates and fillers and the diffusion behavior of the asphalt mastics, which influenced the rheological properties of the asphalt mastics.

## Figures and Tables

**Table 1:** Properties of AH-70 bitumen.

Items	Results	Unit	Requirement
Needle penetration (25 °C)	68.3	0.1 mm	60–80
Ductility (10 °C)	40.1	cm	no less than 25
Ductility (15 °C)	>150	cm	no less than 100
Softening point	48.2	°C	no less than 45
Viscosity (135 °C)	0.450	Pa·s	-
Density (15 °C)	1.035	g/cm^3^	-
Solubility	99.6	%	no less than 99.5

**Table 2:** Relative densities of mineral powders.

**Property**	LP	BP	GP
**Density/g/cm^3^**	2.67	2.87	2.69

**Table 3:** Particle size distributions of three mineral powders.

Items	LP	BP	GP
*D*_10_	2.75	3.27	3.89
*D*_50_	13.08	26.17	26.16
*D*_90_	52.33	62.33	62.23

**Table 4:** Oxide composition of mineral fillers (%).

Oxide	Filler Type		
	LP	BP	GP
CaO	82.828	9.879	2.877
SiO_2_	5.843	45.812	63.87
Al_2_O_3_	4.15	18.552	16.854
Fe_2_O_3_	0.573	11.656	3.116
MgO	4.786	5.954	0.692
K_2_O	0.351	1.893	5.252
Na_2_O	/	2.581	6.023
Other	1.469	3.673	1.316

**Table 5:** Basic performance index of aggregates.

Properties	Limestone	Basalt	Granite	Requirement
Density/g/cm^3^	2.816	3.111	3.070	no less than 2.6
Crushing value/%	23.1	10.2	19.8	no less than 26
Abrasion value/%	20.2	16.6	14.3	no less than 28
Adhesion grade	5	5	3	-

**Table 6:** Specimen designs and names of asphalt mastic–aggregate interfaces.

Type of Interface	Name
Limestone mastic–Limestone	LM–L
Limestone mastic–Basalt	LM–B
Limestone mastic–Granite	LM–G
Basalt mastic–Limestone	BM–L
Basalt mastic–Basalt	BM–B
Basalt mastic–Granite	BM–G
Granite mastic–Limestone	GM–L
Granite mastic–Basalt	GM–B
Granite mastic–Granite	GM–G

**Table 7:** Physical parameters of mineral powders.

Pore Features	LP	BP	GP
Pore volume/cm^3^/g	0.007	0.034	0.009
Average pore size/nm	3.059	3.810	3.819
Surface area/m^2^/g	1.899	9.008	3.42

**Table 8:** Diffusion coefficient of water in different asphalt mastics.

Type of Mastic	Condition	*D*/(cm^2^/s)	*R* ^2^
LP mastic	Water Immersion	1.054 × 10^−10^	0.8396
BP mastic		1.078 × 10^−10^	0.8912
GP mastic		0.938 × 10^−10^	0.8725

## Data Availability

The data presented in this study are available on request from the corresponding author. The data are not publicly available due to confidentiality agreement.
